# Supplementary material for: Unravelling individual rhythmic abilities using machine learning
Source: Sci Rep. 2024 Jan 11;14:1135. doi: 10.1038/s41598-024-51257-7 (PMC10784578; doi:10.1038/s41598-024-51257-7)
Supplement: Supplementary file 1 — Supplementary Information. [file 41598_2024_51257_MOESM1_ESM.pdf]

Supplementary materials

Unravelling individual rhythmic abilities using machine learning

1) List of variables

Full list of variables entered initially in the analysis.

Var #	Variable name	Task	Task category	Outcome measure
1	Dur_discrim_Thresh	Duration discrimination	Perceptual	Threshold for duration discrimination
2	Anisoc_det_tones_Thresh	Anisochrony detection (tones)	Perceptual	Threshold for anisochrony detection with tones
3	Anisoc_det_music_Thresh	Anisochrony detection (music)	Perceptual	Threshold for anisochrony detection with music
4	BAT_all_Dprime	Beat Alignment Test (BAT)	Perceptual	*d'* (sensitivity index) based on all trials (72)
5	BAT_fast_Dprime	Beat Alignment Test (BAT)	Perceptual	*d'* (sensitivity index) based on trials at fast tempo (24)
6	BAT_med_Dprime	Beat Alignment Test (BAT)	Perceptual	*d'* (sensitivity index) based on trials at medium tempo (24)
7	BAT_slow_Dprime	Beat Alignment Test (BAT)	Perceptual	*d'* (sensitivity index) based on trials at slow tempo
8	Unpaced_spont_ITI	Unpaced tapping	Motor	Rate (inter-tap interval) of spontaneous tapping
9	Unpaced_spont_CV	Unpaced tapping	Motor	Variability (coefficient of variation of the inter-tap interval) of spontaneous tapping
10	Unpaced_slow_ITI	Unpaced tapping	Motor	Rate of slow tapping
11	Unpaced_slow_CV	Unpaced tapping	Motor	Variability of slow tapping
12	Unpaced_fast_ITI	Unpaced tapping	Motor	Rate of fast tapping
13	Unpaced_fast_CV	Unpaced tapping	Motor	Variability of fast tapping
14	Paced_metro_450_ITI	Paced tapping (metronome)	Motor	Rate of paced tapping (IOI = 450ms)
15	Paced_metro_450_CV	Paced tapping (metronome)	Motor	Variability of paced tapping (IOI = 450ms)
16	Paced_metro_450_Vector_dir	Paced tapping (metronome)	Motor	Accuracy (vector direction) of paced tapping (IOI = 450ms)
17	Paced_metro_450_Vector_len	Paced tapping (metronome)	Motor	Consistency (vector length) of paced tapping (IOI = 450ms)
18	Paced_metro_600_ITI	Paced tapping (metronome)	Motor	Rate of paced tapping (IOI = 600ms)
19	Paced_metro_600_CV	Paced tapping (metronome)	Motor	Variability of paced tapping (IOI = 600ms)
20	Paced_metro_600_Vector_dir	Paced tapping (metronome)	Motor	Accuracy (vector direction) of paced tapping (IOI = 600ms)
21	Paced_metro_600_Vector_len	Paced tapping (metronome)	Motor	Consistency (vector length) of paced tapping (IOI = 600ms)
22	Paced_metro_750_ITI	Paced tapping (metronome)	Motor	Rate of paced tapping (IOI = 750ms)
23	Paced_metro_750_CV	Paced tapping (metronome)	Motor	Variability of paced tapping (IOI = 750ms)

24	Paced_metro_750_Vector_dir	Paced tapping (metronome)	Motor	Accuracy (vector direction) of paced tapping (IOI = 750ms)
25	Paced_metro_750_Vector_len	Paced tapping (metronome)	Motor	Consistency (vector length) of paced tapping (IOI = 750ms)
26	Paced_music_badine_ITI	Paced tapping (music)	Motor	Rate of paced tapping (music 1, IOI = 600ms)
27	Paced_music_badine_CV	Paced tapping (music)	Motor	Variability of paced tapping (music 1, IOI = 600ms)
28	Paced_music_badine_Vector_dir	Paced tapping (music)	Motor	Accuracy (vector direction) of paced tapping (music 1, IOI = 600ms)
29	Paced_music_badine_Vector_len	Paced tapping (music)	Motor	Consistency (vector length) of paced tapping (music 1, IOI = 600ms)
30	Paced_music_ross_ITI	Paced tapping (music)	Motor	Rate of paced tapping (music 2, IOI = 600ms)
31	Paced_music_ross_CV	Paced tapping (music)	Motor	Variability of paced tapping (music 2, IOI = 600ms)
32	Paced_music_ross_Vector_dir	Paced tapping (music)	Motor	Accuracy (vector direction) of paced tapping (music 2, IOI = 600ms)
33	Paced_music_ross_Vector_len	Paced tapping (music)	Motor	Consistency (vector length) of paced tapping (music 2, IOI = 600ms)
34	Sync_Cont_ 450_ITI	Synchronization-continuation	Motor	Rate of tapping in the continuation phase (IOI = 450ms)
35	Sync_Cont_450_ CV	Synchronization-continuation	Motor	Variability of tapping in the continuation phase (IOI = 450ms)
36	Sync_Cont_450_Motor_variance	Synchronization-continuation	Motor	Motor variance, calculated from tapping in the continuation phase (IOI = 450ms)
37	Sync_Cont_450_Central_variance	Synchronization-continuation	Motor	Central variance, calculated from tapping in the continuation phase (IOI = 450ms)
38	Sync_Cont_600_ITI	Synchronization-continuation	Motor	Rate of tapping in the continuation phase (IOI = 600ms)
39	Sync_Cont_600_ CV	Synchronization-continuation	Motor	Variability of tapping in the continuation phase (IOI = 600ms)
40	Sync_Cont_600_Motor_variance	Synchronization-continuation	Motor	Motor variance, calculated from tapping in the continuation phase (IOI = 600ms)
41	Sync_Cont_600_Central_variance	Synchronization-continuation	Motor	Central variance, calculated from tapping in the continuation phase (IOI = 600ms)
42	Sync_Cont_ 750_ITI	Synchronization-continuation	Motor	Rate of tapping in the continuation phase (IOI = 750ms)

43	Sync_Cont_750_ CV	Synchronization-continuation	Motor	Variability of tapping in the continuation phase (IOI = 750ms)
44	Sync_Cont_750_Motor_variance	Synchronization-continuation	Motor	Motor variance, calculated from tapping in the continuation phase (IOI = 750ms)
45	Sync_Cont_750_Central_variance	Synchronization-continuation	Motor	Central variance, calculated from tapping in the continuation phase (IOI = 750ms)
46	Adaptive_Adapt_index_accel	Adaptive tapping	Motor	Adaptation index (acceleration trials)
47	Adaptive_Adapt_index_decel	Adaptive tapping	Motor	Adaptation index (deceleration trials)
48	Adaptive_Alpha_accel	Adaptive tapping	Motor	Phase correction (alpha coefficient), acceleration trials
49	Adaptive_Alpha_decel	Adaptive tapping	Motor	Phase correction (alpha), deceleration trials
50	Adaptive_Beta_accel	Adaptive tapping	Motor	Period correction (beta coefficient), acceleration trials
51	Adaptive_Beta_decel	Adaptive tapping	Motor	Period correction (alpha), deceleration trials
52	Adaptive_Dprime_min30	Adaptive tapping	Motor	*d'* when perceiving a tempo acceleration (IOI - 30ms)
53	Adaptive_Dprime_min75	Adaptive tapping	Motor	*d'* when perceiving a tempo acceleration (IOI - 75ms)
54	Adaptive_Dprime_plus30	Adaptive tapping	Motor	*d'* when perceiving a tempo deceleration (IOI + 30ms)
55	Adaptive_Dprime_plus75	Adaptive tapping	Motor	*d'* when perceiving a tempo deceleration (IOI + 75ms)

Total number of variables: 55 (7 from perceptual tasks, 48 from motor tasks).

2) Classification of musicians (MUS) and non-musicians (n-MUS)

The confusion matrices for the Train, Validation, and Test sets for the three models (Perc, Motor, PMI) are presented in Table S1, S2, and S3.

Table S1. Confusion matrices for classification based on model Perc.

	Train		Validation		Test	
	Pred n-MUS	Pred MUS	Pred n-MUS	Pred MUS	Pred n-MUS	Pred MUS
Actual n-MUS	13	8	8	2	6	3
Actual MUS	5	19	0	7	1	7

Table S2. Confusion matrices for classification based on model Motor.

	Train		Validation		Test	
	Pred n-MUS	Pred MUS	Pred n-MUS	Pred MUS	Pred n-MUS	Pred MUS
Actual n-MUS	15	6	7	3	8	1
Actual MUS	5	19	0	7	3	5

Table S3. Confusion matrices for classification based on Model PMI.

	Train		Validation		Test	
	Pred n-MUS	Pred MUS	Pred n-MUS	Pred MUS	Pred n-MUS	Pred MUS
Actual n-MUS	15	6	8	2	8	2
Actual MUS	3	21	2	6	0	7

3) Unsupervised learning (modular clustering) for non-musicians

The result of modularity maximization is a partition of non-musicians into two clusters each including 20 participants. This analysis is based on the same variables included in model PMI. The modularity value is $Q = 0.14$. The following Figure shows the heat matrix S with participants in their original order and the same matrix with rows and columns permuted to fit the clusters found above. It is easily seen that intra similarities are stronger (brighter) than inter similarities.

The following Figure shows the heat matrix S with participants in their original order and the same matrix with rows and columns permuted to fit the clusters found above. It can be easily seen that intra-cluster similarities are larger (brighter) than inter-cluster similarities.

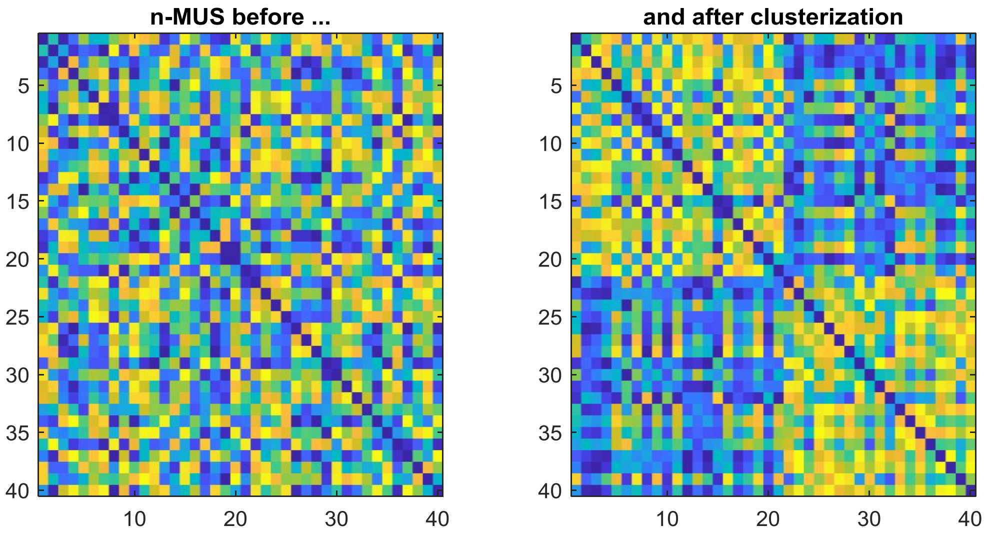

Figure 1. Heat matrices. The brighter the colour the more similar are individuals. Left: Similarity matrix before clustering; Right: the same matrix with rows and columns reordered by clusters C1 and C2.

4) **Classification of the two subgroups of nonmusicians (nMUS-1, nMUS-2) based on the variables included in model PMI**

Table S4. Confusion matrix for classification based on Model PMI.

	Train		Validation		Test	
	Pred nMUS-1	Pred nMUS-2	Pred nMUS-1	Pred nMUS-2	Pred nMUS-1	Pred nMUS-2
Actual nMUS-1	9	0	5	1	5	0
Actual nMUS-2	0	12	0	3	0	5

5) **Classification of the two subgroups of nonmusicians (nMUS-1, nMUS-2) based on the full set of 55 variables (28 variables after PCA)**

An additional clustering analysis was run on the group of 40 non-musicians based on the initial set of 55 variables (see point 1 above). Owing to the relatively small sample size for the large number of variables, the modular clustering algorithm did not converge because the matrix is degenerate. Hence we selected among the variables those which captured most of the variability in the sample by computing the standard deviation: 28 variables (see table below), accounting for 95% of the variance according to a PCA.

Var #	Variable name	Task	Task category
1	Dur_discrim_Thresh	Duration discrimination	Perceptual
2	Anisoc_det_tones_Thresh	Anisochrony detection (tones)	Perceptual
3	Anisoc_det_music_Thresh	Anisochrony detection (music)	Perceptual
4	BAT_all_Dprime	Beat Alignment Test (BAT)	Perceptual
5	Unpaced_spont_ITI	Unpaced tapping	Motor
6	Unpaced_slow_ITI	Unpaced tapping	Motor
7	Unpaced_fast_ITI	Unpaced tapping	Motor
8	Paced_metro_450_ITI	Paced tapping (metronome)	Motor
9	Paced_metro_450_Vector_dir	Paced tapping (metronome)	Motor
10	Paced_metro_600_ITI	Paced tapping (metronome)	Motor
11	Paced_metro_600_Vector_dir	Paced tapping (metronome)	Motor
12	Paced_metro_750_ITI	Paced tapping (metronome)	Motor
13	Paced_metro_750_Vector_dir	Paced tapping (metronome)	Motor
14	Paced_music_badine_ITI	Paced tapping (music)	Motor
15	Paced_music_badine_Vector_dir	Paced tapping (music)	Motor
16	Paced_music_badine_Vector_len	Paced tapping (music)	Motor
17	Paced_music_ross_ITI	Paced tapping (music)	Motor
18	Paced_music_ross_Vector_dir	Paced tapping (music)	Motor
19	Paced_music_ross_Vector_len	Paced tapping (music)	Motor
20	Sync_Cont_ 450_ITI	Synchronization-continuation	Motor
21	Sync_Cont_450_Motor_variance	Synchronization-continuation	Motor
22	Sync_Cont_450_Central_variance	Synchronization-continuation	Motor
23	Sync_Cont_600_ITI	Synchronization-continuation	Motor
24	Sync_Cont_600_Motor_variance	Synchronization-continuation	Motor
25	Sync_Cont_600_Central_variance	Synchronization-continuation	Motor
26	Sync_Cont_ 750_ITI	Synchronization-continuation	Motor
27	Sync_Cont_750_Motor_variance	Synchronization-continuation	Motor
28	Sync_Cont_750_Central_variance	Synchronization-continuation	Motor

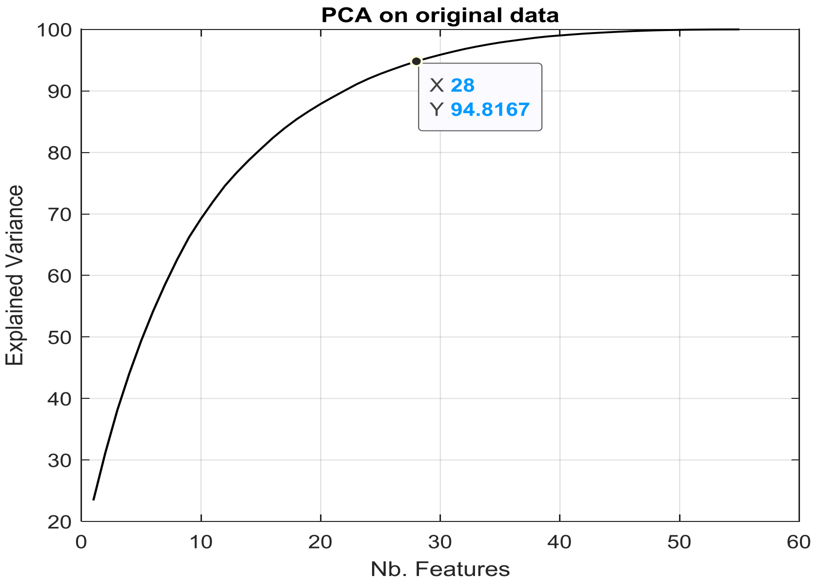

Modular clustering run on these 28 variables provided two clusters within the nonmusicians group, very comparable to those obtained with the profile variables (88% of overlap between the classifications).

References

Hastie T., Tibshirani R., Friedman J., The elements of statistical learning, Springer, 2014.

Tibshirani R. 2011. Regression shrinkage and selection via the Lasso: a retrospective. Journal of the Royal Statistical Society: Series B 73:273-282.

Combettes P. L., Pesquet J. C., A Douglas-Rachford splitting approach to non-smooth convex variational signal recovery. IEEE Trans. Signal Process., 1(4), 2007.

Jiu M., Pustelnik N., Janaqi S., Multiclass svm with graph path coding regularization for face classification, IEEE on Machine Learning for Signal Processing, 2016, Salerno, Italy/

Jiu M., Pustelnik N., Janaqi S., Sparse hierarchical interaction learning with epigraphical projection, arXiv, decembre 2017.

Faul F, Erdfelder E, Buchner A, Lang A-G. 2009. Statistical power analyses using G*Power 3.1: Tests for correlation and regression analyses. Behavior Research Methods 41:1149-1160. Doi: 10.3758/BRM.41.4.1149

Azen R, Budescu DV. 2003. The dominance analysis approach for comparing predictors in multiple regression. Psychological Methods 8:129–148. doi: 10.1037/1082-989X.8.2.129, PMID: 12924811

Newman M. E. J., Modularity and community structure in networks. PNAS June 6, 2006 103 (23) 8577-8582; https://doi.org/10.1073/pnas.0601602103.

Rubinov, M., Sporns, O., 2010. Complex network measures of brain connectivity: Uses and interpretations. Neuroimage 52, 1059–1069. doi:10.1016/j.neuroimage.2009.10.003
